# HotBalloon ablation of atrial fibrillation in patients with dextrocardia and situs inversus by “mirror image” approach

**DOI:** 10.1002/joa3.12240

**Published:** 2019-10-22

**Authors:** Shunsuke Miyauchi, Yukiko Nakano, Yoshihiro Ikeuchi, Sho Okamura, Yousaku Okubo, Naoya Hironobe, Takehito Tokuyama, Yasuki Kihara

**Affiliations:** ^1^ Department of Cardiovascular Medicine Graduate School of Biomedical and Health Sciences Hiroshima University Hiroshima Japan

**Keywords:** ablation, atrial fibrillation, dextrocardia, hotballoon, mirror image

## Abstract

A 51‐year‐old man with dextrocardia and complete situs inversus who presented with palpitation as a result of drug refractory paroxysmal atrial fibrillation (AF) was referred to our hospital for catheter ablation. Pulmonary vein isolation (PVI) was performed using the HotBalloon technique. We were able to accomplish AF ablation safely and easily using 180 degree‐inversed mirror images of X‐ray fluoroscopy. We have reported successful PVI for AF with dextrocardia using HotBalloon and mirror image of X‐ray fluoroscopy without any complication for the first time.

## Graphical abstract

We report a case of successful pulmonary vein isolation for atrial fibrillation with dextrocardia using HotBalloon and 180 degree‐inversed mirror image of X‐ray fluoroscopy for the first time.

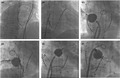

## 1 INTRODUCTION

The frequency of dextrocardia with situs inversus was reported as 1–2/20000 in the general population.^1^ Pulmonary vein isolation (PVI) for atrial fibrillation (AF) in patients with dextrocardia is challenging. A few reports have demonstrated successful PVI in patients with dextrocardia and situs inversus using radiofrequency ablation or cryoballoon ablation.^2^, ^3^ The HotBalloon technique has also been established as a simple technique for PVI for AF.^4^ We report successful PVI for AF with dextrocardia using the HotBalloon technique for the first time.

## 2 CASE REPORT

**Figure 1:**
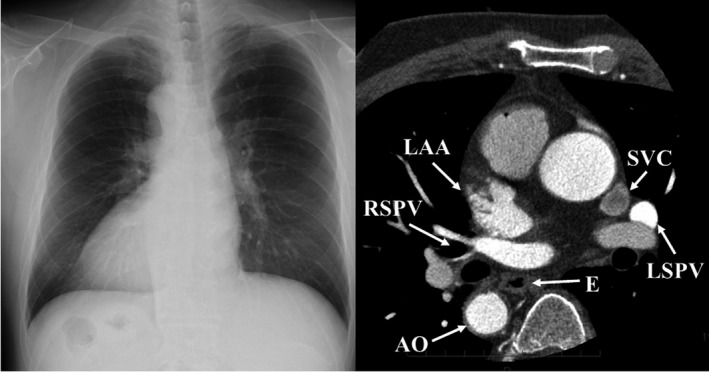
Chest X‐ray and computed tomography showing dextrocardia with complete situs inversus without any other anatomical complications. AO, aorta; E, esophagus; LAA, left atrial appendage; LSPV, left superior pulmonary vein; RSPV, right superior pulmonary vein; SVC, superior vena cava

**Figure 2:**
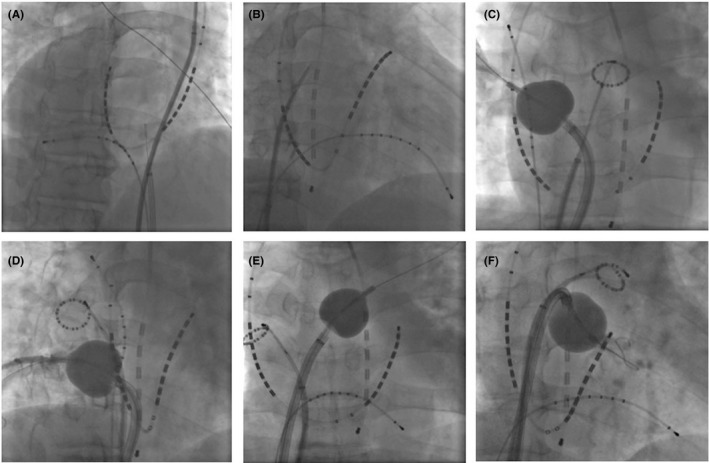
A and B, X‐ray fluoroscopic images in right anterior oblique. The X‐ray fluoroscopic image is reversed by 180° to obtain the mirror image, and the procedure can be performed without any confusion in the orientation. C‐F, The usual order of PVI by HotBalloon was performed (1. left superior PV, 2. left inferior PV, 3. right superior PV, and 4. right inferior PV)

A 51‐year‐old man with dextrocardia and situs inversus who presented with palpitation due to drug refractory paroxysmal AF was referred to our hospital for catheter ablation. Preprocedural three‐dimensional computed tomography (3D CT) revealed complete situs inversus without any other anatomical complications (Figure 1). We selected the HotBalloon technique as a strategy for PVI. An intracardiac defibrillation catheter (BeeAT, Japan Lifeline) was inserted into the coronary sinus via the left internal jugular vein. All other venous access sites were achieved from the right femoral vein (Figure 2A). We attempted to perform transseptal puncture under intracardiac echocardiography (ViewFlex, St. Jude Medical) using a transseptal needle (RF Needle, Japan Lifeline) through an 8.0‐Fr transseptal sheath (SL1, St. Jude Medical). Before transseptal puncture, we reversed the X‐ray fluoroscopic image by 180° to obtain a mirror image by changing the setup of the interventional angiography system (Artis Q, Siemens Healthineers) (Figure 2B). To obtain a mirror image, we performed the procedure with the same orientation of fluoroscopic image as usual, while with completely opposite counterclockwise direction of the catheter. The 8.0‐Fr transseptal sheath was exchanged to a deflectable guiding sheath (Treswaltz, Toray Industries) and the Satake HotBalloon ablation system (Toray Industries) was inserted into the left atrium. The HotBalloon was placed on each ostium and carina of the pulmonary vein (PV) by advancing the guidewire (Spring Guide Wire, Toray Medical). The appropriate balloon size for each PV was obtained by adjusting the saline volume injected to the balloon lumen. The usual order of PVI was performed with one balloon for each PV (1. left superior PV ostium, 2. left inferior PV, 3. right superior PV, and 4. right inferior PV) (Figure 2C‐F). Because of complete situs inversus, the positional relationship between the four PVs, esophagus, and phrenic nerve was equal to that in usual cases on the mirror fluoroscopic image. The temperature of the esophagus was continuously monitored, particularly during left PVI using an esophageal temperature monitoring system (Esophaster, Japan Lifeline). Continuous monitoring of the phrenic nerve was also performed by using an electrode placed in the superior vena cava. After HotBalloon ablation in the four PVs, entrance and exit blocks were confirmed in the right superior, right inferior, and left inferior PVs. Electrical isolation of the left superior PV could not be achieved by additional HotBalloon ablation, and touch‐up ablation to the antrum ridge by a radiofrequency catheter was required (FlexAbility, St. Jude Medical). Finally, we achieved successful PVI without any complications. The total fluoroscopic time was 51 minutes and the total procedural time was 197 minutes in this case, while the average required total fluoroscopic time and procedural time to achieve successful PVI using HotBalloon in the other cases were 63 and 141 minutes in our institution, respectively. After the procedure, the patient was free from an AF episode during the 12‐month follow‐up period, without administration of any antiarrhythmic agents.

## 3 DISCUSSION

This case is unique based on the following points: First, to our knowledge, this is the first report on successful HotBalloon ablation in AF with dextrocardia and situs inversus. Second, we used a mirror image of X‐ray fluoroscopy during ablation, which made the procedure easy and safe.

The frequency of dextrocardia with situs inversus was reported as 1–2/20000 in the general population,^1^ and successful PVI for these patients has been achieved by radiofrequency catheter ablation in a few reports.^2^ Recently, Yoshiga et al^3^ reported a case of cryoballoon ablation in patients with paroxysmal AF with dextrocardia and situs inversus. Similarly with cryoballoon, the HotBalloon technique is a safer and easier method and has been established as a strategy for ablation of paroxysmal AF.^4^ Moreover, compared with other techniques, the HotBalloon technique may be superior in deforming to various sizes and shapes corresponding to the shapes of the PVs. Therefore, we preferred to use the HotBalloon technique.

We used the mirror image of X‐ray fluoroscopy. Serkan et al^5^ succeeded in accessory pathway ablation of Wolff–Parkinson–White disease in a patient with complete situs inversus by mirror image approach. We first demonstrated the usefulness of the mirror image approach in PVI performed using the balloon techniques. This approach enabled us to achieve successful HotBalloon ablation with the same orientation of fluoroscopic image without any special care compared to usual procedures, while a completely opposite counterclockwise direction was required in case of dextrocardia with complete situs inversus. Confirmation of the existence of any other anatomical complications by preprocedural echocardiography and 3D CT is needed in such a case.

In the selection of a strategy for a case with dextrocardia, an easy‐to‐use approach and a mirror fluoroscopic image is useful to accomplish the procedure.

## CONFLICT OF INTEREST

Authors declare no conflict of interests for this article.
